# ULK3-dependent activation of GLI1 promotes DNMT3A expression upon autophagy induction

**DOI:** 10.1080/15548627.2022.2039993

**Published:** 2022-02-28

**Authors:** Patricia González-Rodríguez, Mathilde Cheray, Lily Keane, Pinelopi Engskog-Vlachos, Bertrand Joseph

**Affiliations:** a Institute of Environmental MedicineToxicology Unit, Karolinska Institutet, Stockholm Sweden; b Division of Biochemistry, Department of Molecular Medicine, Institute of Basic Medical Sciences, University of Oslo, Oslo, Norway

**Keywords:** Autophagy, DNMT3A, GLI1, transcription, ULK3

## Abstract

Macroautophagy/autophagy is a tightly regulated catabolic process, which contributes at baseline level to cellular homeostasis, and upon its stimulation to the adaptive cellular response to intra- and extracellular stress stimuli. Decrease of autophagy activity is occurring upon aging and thought to contribute to age-related-diseases. Recently, we uncovered, upon autophagy induction, the role of *de novo* DNMT3A (DNA methyltransferase 3 alpha)-mediated DNA methylation on expression of the MAP1LC3 (microtubule associated protein 1 light chain 3) proteins, core components of the autophagy pathway, which resulted in reduced baseline autophagy activity. Here, we report that serine/threonine kinase ULK3 (unc-51 like kinase 3)-dependent activation of GLI1 (GLI family zinc finger 1) contributes to the transcriptional upregulation of *DNMT3A* gene expression upon autophagy induction, thereby bringing additional understanding of the long-term effect of autophagy induction and a possible mechanism for its decline upon aging, pathological conditions, or in response to treatment interventions.

**Abbreviations:** CBZ: carbamazepine; ChIP: chromatin immunoprecipitation; Clon: clonidine; DNMT3A: DNA methyltransferase 3 alpha; GLI1: GLI family zinc finger 1; GLI2: GLI family zinc finger 2; MAP1LC3: microtubule associated protein 1 light chain 3; MTOR: mechanistic target of rapamycin kinase; PLA: proximity ligation assay; RT-qPCR: quantitative reverse transcription PCR; shRNA: small hairpin RNA; siRNA: small interfering RNA; Treh: trehalose; ULK3: unc-51 like kinase 3.

## Introduction

Macroautophagy (hereafter referred as autophagy) is a conserved catabolic pathway in which portions of the cytoplasm like protein aggregates, damaged organelles or pathogenic organisms are targeted for degradation. This form of autophagy involves double membrane-rearrangements, called autophagosomes, which surrounds a portion of the cytoplasm that eventually fuses with the lysosomes for the degradation and recycling of their cargo [1]. Under physiological conditions, a basal level of this process is required to maintain cellular homeostasis [2–4]. In addition, this process can be enhanced to respond to a range of extra- or intracellular stress conditions thereby serving an adaptive role to protect organisms [5,6]. Consequently, disturbances in this biological process have been linked to many human diseases, including neurodegenerative diseases, metabolic disorders, psychiatric disorders, cardiovascular diseases and cancers [3,5,7].

Autophagy is a tightly regulated process in both the cytoplasmic and the nuclear cell compartments. In the cytoplasm, the series of dynamic-rearrangements are orchestrated by a core set of autophagy-related proteins [1,8]. In the nucleus, transcription factors as well as chromatin modifying enzymes have gained interest in the transcriptional and epigenetic regulation of autophagy, respectively [^9–12^]. Indeed, accumulating evidence revealed a role for histone modifications in the short-term transcriptional response to stimuli eliciting autophagy [^13–17^]. Moreover, we recently uncovered a role for DNA-methylation in the long-term transcriptional control of autophagy. In fact, we report that autophagy induction is associated with an upregulation of the expression of the *DNMT3A* (DNA methyltransferase 3 alpha) gene. DNMT3A protein was found to be recruited to and promote the methylation of the promoter region of *MAP1LC3* genes encoding for microtubule-associated protein 1 light chain 3 isoforms, which are essential core components of the autophagic machinery. The consequent persistent transcriptional downregulation of MAP1LC3 protein expression negatively affected autophagy capacity of the cells [18]. In this brief report, we aimed at the identification of the signaling pathway that could mediate the regulation of *DNMT3A* gene expression upon autophagy induction.

## Results

### GLI1 is upregulated, phosphorylated, translocated to the nucleus and recruited to *DNMT3A* promoter upon autophagy induction

There is compelling evidence that several transcription factors play essential roles in the regulation of the autophagy process [6,13,19]. Based on a literature search for transcription factors, whose expression/activity has been shown to be modulated upon autophagy induction and which have the ability to directly affect *DNMT3A* gene transcription, GLI1/Glioma-associated oncogene homolog 1 (GLI family zinc finger 1) was identified as a potential transcription factor candidate for the control of autophagy-induced upregulation of *DNMT3A* gene expression [20].

**Figure 1.**
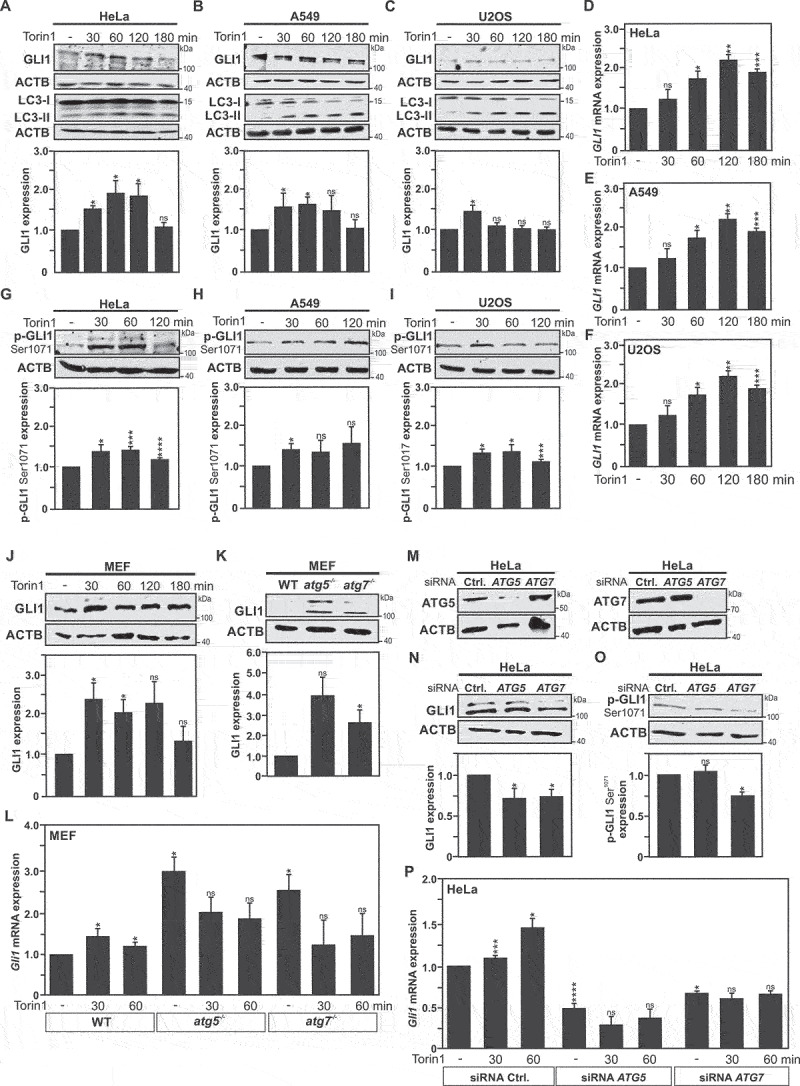
Autophagy induction dependent GLI1 upregulation and phosphorylation at Serine 1071 residue. Immunoblot analysis of GLI1, LC3-I and LC3-II expression in (A) HeLa cells, (B) A549 cells and (C) U2OS cells upon Torin1 treatment at 30-, 60-, 120- and 180-min timepoints reveal an increase in GLI1 protein expression upon autophagy induction. DMSO (used as solvent)-treated cells were used as controls for all experiments performed with Torin1 treatment. (A-C) The graphs show quantification of GLI1 versus ACTB expression in respective cell lines. RT-qPCR analysis of *GLI1* mRNA expression in (D) HeLa cells, (E) A549 cells and (F) U2OS cells upon same treatment as above. Analysis of GLI1 phosphorylation at serine 1071 residue (p-GLI1 Ser1071) by immunoblotting after 30-, 60-, and 120-min Torin1 treatment indicates increased GLI1 phosphorylation upon autophagy induction in (G) HeLa cells, (H) A549 cells and (I) U2OS cells. (G-H) The graphs display the quantification of p-GLI1 Ser1071 versus ACTB expression. (J) Immunoblot analysis of GLI1 expression in MEF cells upon Torin1 treatment at 30, 60, 120 and 180 min timepoints reveal an increase in GLI1 protein expression upon autophagy induction. (K) Immunoblot analysis of baseline GLI1 expression in wild-type (WT), *atg5*^−/−^ and *atg7*^−/−^ MEF cells. (J-K) The graph shows quantification of GLI1 versus ACTB expression. (L) *Gli1* mRNA expression level analyzed by RT-qPCR upon autophagy induction with Torin1 at 30 and 60 min in WT, *atg5*^−/−^ and *atg7*^−/−^ MEF cells. (K-L) Comparisons were performed between autophagy deficient MEF cells (*atg5*^−/−^ and *atg7*^−/−^) and WT MEF cells. (M-P) HeLa cells were transfected with siRNAs pool targeting *ATG5* or *ATG7* expression. (M) Immunoblot analysis of ATG5 and ATG7 expression validates successful siRNA-mediated *ATG5* or *ATG7* silencing in HeLa cells. Immunoblot analyses and quantifications of GLI1 (N) and p-GLI1 Ser1071 expression (O) versus ACTB expression in HeLa cells under the above-described condition reveal decreased expression in autophagy-deficient HeLa cells. (P) Analysis of *GLI1* mRNA by RT-qPCR reveals decreased expression in HeLa cells transfected with *ATG5* or *ATG7* siRNA pools but no significant changes upon autophagy induction with Torin1 in these cells, compared to mock transfected HeLa cells used as control ones, which revealed an increase of *GLI1* expression upon autophagy induction. All values are means of at least 3 independent experiments ± SEM and considered significant for *p < 0,05, **p < 0,01, ***p < 0,001 and ****p < 0,0001. n.s, not significant for the indicated comparison. (**A**, n = 6; **B**, n = 5; **C**, n = 4; **D-F**, n = 4; **G-I**, n = 3; **J-L**, n = 4; **M-P**, n = 3).

In fact, an upregulation of GLI1 expression levels, upon autophagy induction, was observed in endocervical adenocarcinoma HeLa, lung adenocarcinoma A549, and osteosarcoma U2OS cancer cells, as early as 30 minutes post treatment with Torin1 (MTOR-dependent inducer of autophagy) (Figure 1A-C), or nutrient starvation (**Fig. S1A-C**). Likewise, treatment of HeLa cells, with MTOR-independent (carbamazepine, trehalose and clonidine) inducers of autophagy resulted in increased GLI1 expression levels (**Fig. S2D**). Of note, among this panel of autophagy inducers, carbamazepine was found to be the least efficient in promoting an increase of GLI1 expression. Moreover, analysis of *GLI1* mRNA levels by RT-qPCR, revealed a significant upregulation of the messenger upon autophagy induction in HeLa, A549 and U2OS cells, indicative of transcriptional regulation of GLI1 expression, responsible for the observed increases at protein level (Figure 1D-F**, and Fig. S1D-F**).

Furthermore, in order to ascertain that GLI1 induction is dependent on the autophagy process, experiments were performed with *atg7*^−/−^ and *atg5*^−/−^ mouse embryonic fibroblasts (MEF), *i.e*. autophagy deficient cells. Immunoblotting and RT-qPCR analyses revealed that both *atg5-* and *atg7*-deficiencies in MEF cells are associated with a significant increase in *Gli1*/GLI1 expression at both mRNA and protein levels in baseline conditions (Figure 1K-L). Hence, it appears that autophagy deficiency may have an impact on the turnover of GLI1 in the autophagy deficient MEF cells. It can be speculated that both the absence of autophagy dependent-GLI1 degradation and transcriptional compensatory mechanism that would increase *Gli1* gene expression can contribute to the observed effect on *Gli1*/GLI1 expression levels. In fact, the iLIR (in silico identification of functional LC3 Interacting Region Motifs) bioinformatic tool reveals that human and mouse GLI1 proteins contain a conserved predicted LIR motif (LDFVAI, residues 1047 to 1052 in the human protein, and 1052 to 1057 in the murine protein) [21,22].Transcriptional compensatory mechanisms are reported in the context of prolonged autophagy induction and autophagy deficiency [^23–27^]. However, upon autophagy induction no further increase in *Gli1* mRNA expression was observed in *atg7*^−/−^ and *atg5*^−/−^ MEFs cells, suggesting that autophagy deficiency abrogates the ability to promote enhance *Gli1*/GLI1 expression (Figure 1L). In contrast, wild-type MEF cells, like the panel of cancer cells tested, exhibit an increase in GLI1 expression when challenged with starvation or Torin1 treatment (Figure 1J **and Fig. S1J**). An additional set of experiments were performed with HeLa cells transfected with a pool of siRNAs directed against *ATG5* or *ATG7* (Figure 1M), which efficiently decreased the expression of GLI1 in these cells (Figure 1N). Corroborating the observation made in MEF cells, *GLI1* mRNA expression was found to be increased at 30 and 60 min after Torin1 treatment in control cells (*i.e*. CTRL-siRNA HeLa), whereas in *ATG5*-siRNA and *ATG7*-siRNA transfected HeLa cells *GLI1* mRNA levels were not found to be significantly affected upon autophagy induction (Figure 1P). Collectively, these data indicate a regulation of GLI1 expression upon autophagy induction.

GLI proteins transcriptional activities are controlled by post-translational modifications via phosphorylation at multiple sites, which either activate or inactivate these transcription factors [28]. All GLI family members harbor a highly conserved zinc-finger DNA binding domain, which contains activating phosphorylation sites followed by a nuclear localization sequence that regulate its cellular functions and subcellular localization. However, whereas GLI2/3 contain a shared repressor domain and associated regulatory post-translational modifications, GLI1 lacks this repressor domain and thus post-translational modifications by phosphorylation of serine/threonine residues only acts as a transcriptional activator of GLI1 [29,30]. Hence, we decided to investigate whether activating phosphorylation of GLI1 can be observed upon autophagy induction. Taking advantage of an antibody directed against phospho-GLI1 (Ser1071), increased phosphorylation of GLI upon Torin1- or starvation-induced autophagy was confirmed by immunoblotting in HeLa, A549 and U2OS cells (Figure 1G-I **and Fig. S1G-I**). Conversely, phosphorylation of GLI levels were found to be significantly decreased in *ATG7*-siRNA transfected HeLa cells, which brings an additional indication of the role of autophagy on the regulation of GLI1 activation and thus, its consequent functions (Figure 1O).

**Figure 2.**
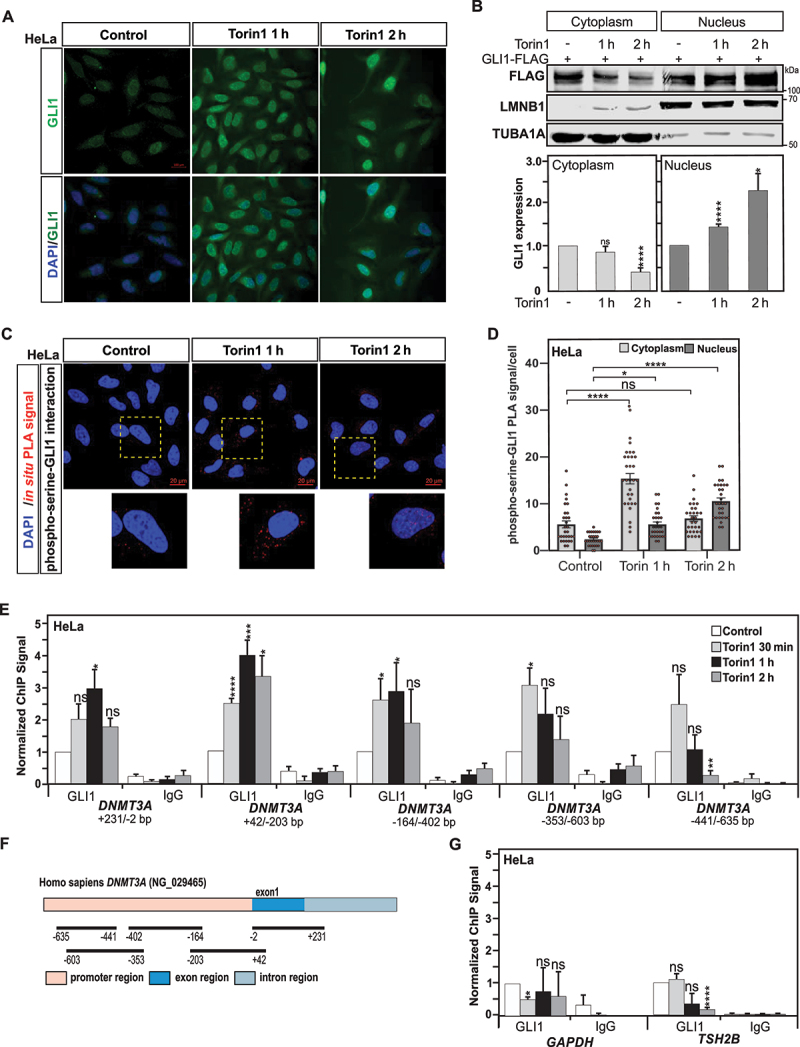
GLI1 translocates to the nucleus and is recruited to *DNMT3A* promoter upon autophagy induction. (A) Representative immunofluorescence confocal images for GLI1, using DAPI for nuclear counterstaining, show that GLI1 is upregulated and displays a nuclear accumulation after 1- and 2-h treatment with Torin1 in HeLa cells. Scale: 100 µm. (B) Immunoblot analysis of the cytoplasmic and nuclear cell extracts from GLI1-Flag transfected HeLa cells confirmed GLI1 nuclear accumulation upon autophagy induction after Torin1 treatment for 1 and 2 h. (C) Phosphorylation of GLI1 at serine residues was assayed by *in situ* proximity ligation assay (PLA) in HeLa cells treated with Torin1 for 1 or 2 h Nuclear counterstaining with DAPI was used to evaluate nuclear versus cytoplasmic localization of the phospho-serine-GLI1 interactions. Scale: 20 µm. (D) Quantification of the *in situ* PLA experiments displayed in panel C. Statistics were performed with a one-way ANOVA (Pairwise Multiple Comparison by the Holm-Sidak method), n = 30 and 95% confidence intervals are shown. (E) Chromatin immunoprecipitation (ChIP) analysis of GLI1 recruitment at different *DNMT3A* promoter and exon 1 regions show GLI1 enrichment upon induction of autophagy with Torin1 treatment in HeLa cells. (F) Schematic representation of regions within promoter and exon 1 of *DNMT3A* locus analyzed for GLI1 ChIP (localization of regions are indicated in base pairs (bp) in relation to the transcription start site (TSS). (G) No significant GLI1 enrichments were observed at *GAPDH* and *TSH2B* loci used as GLI1-nonbinding regions and negative controls. All values are means of at least 3 independent experiments ± SEM and considered significant for *p < 0,05, **p < 0,01, ***p < 0,001 and ****p < 0,0001. n.s, not significant for the indicated comparison. (**A**, n = 3; **B**, n = 3; **C-D**, n = 3; **E** and **G**, n = 4).

GLI1 protein shuttling between the cytoplasm and nucleus is tightly associated with the regulation of its function as a transcription factor [31]. Under steady state conditions, GLI1 is found to be chiefly present in the cytoplasm, but nuclear localization is also reported [32,33]. However, upon specific cellular stimuli that trigger its activation, such as its phosphorylation, GLI1 is found to translocate and accumulate in the nucleus, where it can regulate the expression of target genes [34]. Therefore, the subcellular localization of GLI1 in HeLa cells was investigated upon autophagy induction. Immunofluorescence analysis showed that under control conditions, GLI1 appeared to be localized in both the cytoplasm and nucleus, preferentially located in the perinuclear area (Figure 2A). In agreement with the immunoblot data, after 1 h treatment with Torin1 a significant increase in GLI1 protein expression was observed. Furthermore, a nuclear accumulation of GLI1, sustained up to 2 h post Torin1 treatment, was also observed upon autophagy induction (Figure 2A). Moreover, in GLI1-FLAG tagged expressing HeLa cells, subcellular fractionation and immunoblotting further confirmed the cytoplasm to nucleus translocation of GLI1 upon autophagy induction (Figure 2B).

*In situ* Proximity Ligation Assay (PLA) is commonly used to detect protein-protein interactions but the detection of various protein posttranslational modifications has also emerged as one of its applications [35,36]. Of cautiousness, before using an *in situ* PLA to identify proteins with phospho-serine residues, we first gained direct evidence by immunoblotting analysis that GLI1 is indeed phosphorylated upon autophagy induction. Thus, taking advantage of the *in situ* PLA for detection of phospho-serine residues-GLI1 interactions, we found an increased phosphorylation of GLI1 in HeLa cells exposed to nutrient starvation, and treatments with MTOR-dependent and -independent inducers of autophagy (Figure 2C-D **and Fig. S2A and C**). Nuclear accumulation of phosphorylated GLI1 was also observed upon autophagy induction under the above-described conditions (Figure 2C-D **and Fig. S2A and C**).

Finally, to gain direct evidence that GLI1 regulates *DNMT3A* gene expression under autophagy conditions, the recruitment of this transcription factor to the promoter region of *DNMT3A* was investigated. For this purpose, chromatin immunoprecipitation (ChIP) of GLI1 was performed for different overlapping regions covering part of the promoter region and exon1 of the *DNMT3A* gene (Figure 2E-F). This analysis of GLI1 chromatin occupancies reveals that Torin1-induced, as well as starvation-induced autophagy in HeLa cells is associated at 30 min with a selective GLI1 enrichment in the regions closer to the Transcription Start Site (TSS) of the *DNMT3A* gene (Figure 2E-F**, and Fig. S1K**). Noteworthy, this GLI1 recruitment appears to be transient, as illustrated by the decreased GLI1 occupancy observed already at 2 h post-treatment. Collectively these data indicate that the activation of transcription factor GLI1 contributes to the upregulation of *DNMT3A* gene expression observed upon autophagy induction.

### ULK3 kinase activity mediates GLI1 activation upon autophagy induction

Thereafter, we set out to identify which kinase might be activated upon autophagy induction and be responsible for the phosphorylation and activation of GLI1 leading to *DNMT3A* gene expression. Interestingly, the serine/threonine kinase ULK3 (unc-51 like kinase 3), can phosphorylate GLI1, promoting its nuclear localization and enhancing its transcriptional activity, and should therefore be considered as a positive regulator of GLI1 [37]. In contrast, under similar conditions, ULK1 was reported to be unable to increase GLI1 transcriptional activity [37]. Whereas a role for ULK1 and ULK2, mammalian homolog of the yeast Atg1, and somehow redundant kinases, in the regulation of autophagy is well established [38], the function of ULK3 in this process remains unclear. ULK1 and ULK2 share significant domain similarities, that are not restricted to their kinase domain (79% identity), as the two proteins share an intrinsically disordered region (IDR) and a carboxy-terminal domain (CTD)-like domain (essential for the recruitment of ATG13) [39,40]. However, the IDR and CTD-like domains are both absent in ULK3, which also share only 40% sequence identity in its kinase domain with ULK1 and ULK2 [39]. In addition, ULK3 is reported to be upregulated upon autophagy induction by serum starvation or amino acid depletion [41,42]. Some reports also indicate that ULK3 expression *per se* can play a role in the initiation of autophagy [41,43]. These observations urged us to investigate whether ULK3 could be involved in the regulation of GLI1 activation upon autophagy induction.

**Figure 3.**
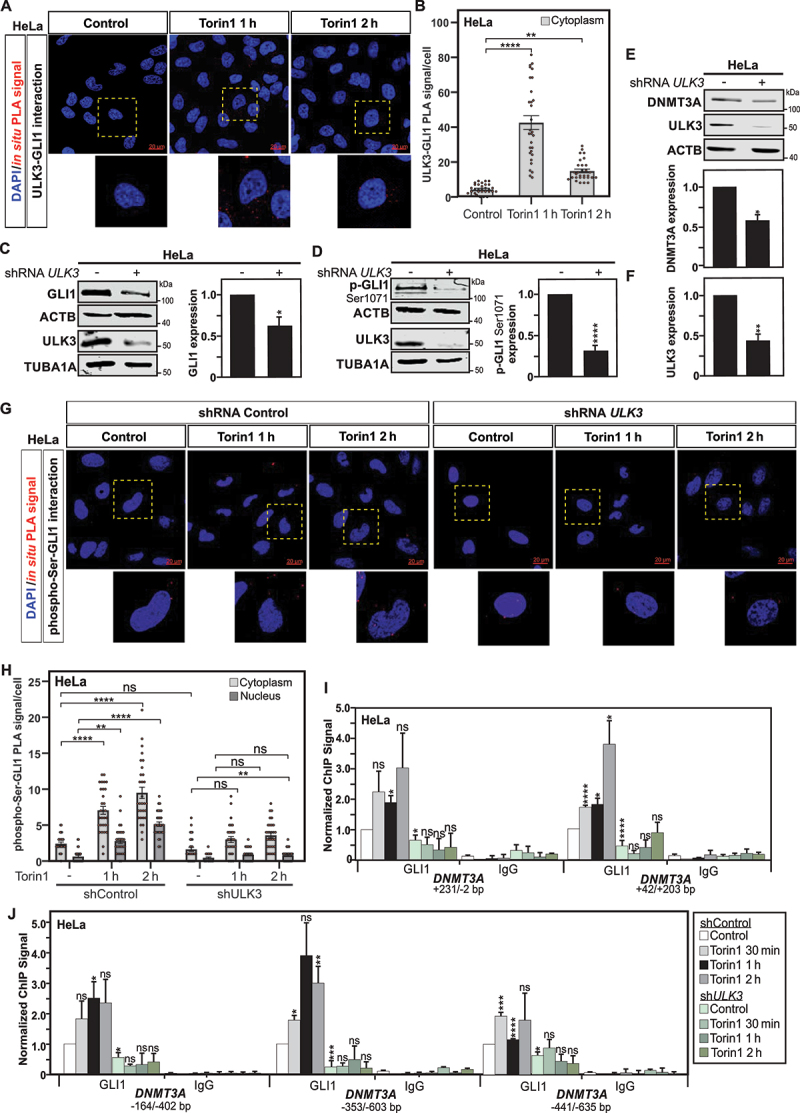
ULK3 mediates GLI1 activation and phosphorylation. (A) *in situ* PLA assay reveals protein interaction between ULK3 and GLI1 in HeLa cells treated with Torin1 for 1 or 2 h. DAPI was used for nuclear counterstaining. Scale: 20 µm. (B) Quantification of the *in situ* PLA experiments displayed in panel A. Statistics were performed with a one-way ANOVA (Pairwise Multiple Comparison by the Holm-Sidak method), n = 30 and 95% confidence intervals are shown. (C) Immunoblot analysis and quantification of GLI1 and ULK3 expression show reduced expression for both proteins in shRNA *ULK3* transduced HeLa cells as compared to shRNA control transduced ones. ACTB and TUBA1A were used as loading controls for GLI1 and ULK3, respectively. The graph shows the quantification for GLI1 *versus* ACTB expression. Under the same conditions, immunoblot analyses and quantifications of p-GLI1 Ser1071 (D) and DNMT3A (E), both normalized with ACTB expression, illustrate decreased GLI1 phosphorylation as well as DNMT3A expression levels in shRNA *ULK3* HeLa cells, as compared to shRNA control cells, respectively. (F) Quantification of ULK3 confirms the successful repression of ULK3 expression in HeLa transduced shRNA *ULK3* as compared to cells transduced with shRNA control. (G) Analysis of phosphorylation of GLI1 at serine residues by *in situ* PLA revealed reduced GLI1 phosphorylation as well as nuclear localization in Torin1 treated shRNA *ULK3* transduced HeLa cells as compared to their shRNA control transduced counterpart. Nuclear counterstaining with DAPI was used to evaluate nuclear versus cytoplasmic localization of the phospho-serine-GLI1 interactions. Scale: 20 µm. (H) Quantification of phospho-serine and GLI1 interactions per cell of experiments displayed in panel G. Statistics were performed using two-way ANOVA for multiple comparisons between treatments and both cell types. n = 30 and 95% confidence intervals are shown. (I-J) Analysis of GLI1 recruitment to *DNMT3A* promoter and exon 1 regions by ChIP reveals an absence of GLI1 enrichment at *DNMT3A* locus in shRNA *ULK3* HeLa cells upon Torin1 treatment, as compared to shRNA control HeLa cells where significant GLI1 recruitment is observed upon autophagy induction. GLI1 ChIP for the *GAPDH* and *TSH2B* loci used as negative controls are depicted in supplementary figure S3 panel A. All values are means of at least 3 independent experiments ± SEM and considered significant for *p < 0,05, **p < 0,01, ***p < 0,001 and ****p < 0,0001. n.s, not significant for the indicated comparison. (**A**, n = 3; **C-D**, n = 3; **E** and **G**, n = 4).

Therefore, two different sets of experiments using *in situ* PLA were performed to examine i) whether protein-protein interaction between ULK3 and GLI1 is increased upon autophagy induction and ii) whether ULK3 deficiency could have an impact on the phosphorylation of GLI1 under these conditions. A notable increase in the number of interactions, as assayed by *in situ* PLA, between ULK3 and GLI1 proteins upon induction of autophagy was observed upon starvation or treatment with both MTOR-dependent and MTOR-independent inducers of autophagy (Figure 3A-B**, Fig. S2A-B and Fig. S2E-F**). The validation that the interaction of GLI1 with the ULK3 kinase is associated with the phosphorylation and activation of this transcription factor including its translocation to the nucleus. GLI1 phosphorylation and subcellular localization was illustrated by *in situ* PLA, as previously described (Figure 2C-D**, Fig. S2A and C, and Fig. S2E and G**).

To further support the involvement of ULK3 in this process, its expression was repressed using a small hairpin RNA (shRNA) targeting *ULK3* gene expression (Figure 3C-H). In fact, the effective downregulation of ULK3 expression in HeLa cells monitored by immunoblot analyses was found to effectively affect GLI1 expression, as well as its phosphorylation at Ser1071 residue (Figure 3C-D and F). However, possible redundancy with ULK1 and ULK2 was noted as pools of siRNAs designed to target the expression of *ULK1* or *ULK2* were found to efficiently reduce phosphorylated GLI1 expression level in HeLa cells (**Fig. S2H-I**). *In situ* PLA further confirmed the reduction of phosphorylation of GLI1 at serine residues, as well as illustrated reduced nuclear subcellular localization of phosphorylated GLI1, upon autophagy induction by Torin1 treatment, in shRNA *ULK3* transduced HeLa cells as compared to shRNA control transduced ones (Figure 3 G-H). Furthermore, in order to gain additional evidence for a role of ULK3 in the regulation of GLI1-control of *DNMT3A* gene expression, the effect of shRNA-mediated knockdown of *ULK3* on DNMT3A expression *per se* was examined in HeLa cells. In agreement with the proposal that ULK3-dependent GLI1 activation indeed promotes *DNMT3A* gene expression, repression of *ULK3* gene expression in HeLa cells was associated with decreased DNMT3A protein expression (Figure 3E-F). ChIP analysis also revealed under these conditions that repression of ULK3 is associated with reduced recruitment of GLI1 to the *DNMT3A* promoter (Figure 3I-J **and Fig. S3A**).

Finally, to obtain additional evidence that ULK3 kinase activity affects the phosphorylation status of GLI1 and thus its activation, we took advantage of expression vectors encoding for Flag-tagged versions of wild-type ULK3, and for point mutated ULK3^K139R^, which corresponds to a catalytically inactive version of ULK3. Remarkably, expression of mutated ULK3^K139R^ in HeLa cells negatively affected on the expression levels of GLI1 and phosphorylated GLI1 (Ser1071) investigated by immunoblotting (**Fig. S3B and S3C**). Immunofluorescence analysis for GLI1 confirmed the reduced GLI protein expression (**Fig. S3D**). Subcellular fractionation and immunoblotting for the FLAG-tagged GLI1, indicated an absence of relocalization of GLI1 to the nucleus upon Torin1-induced autophagy in HeLa expressing the catalytically inactive version of ULK3, as compared to cells expressing its wild-type version (**Fig. S3E**). This finding was also illustrated by GLI1 immunofluorescence analysis (**Fig. S3D**).

### ULK3- and GLI1-dependent control of *DNMT3A* expression is required for downregulation of *MAP1LC3* expression observed upon autophagy induction

**Figure 4.**
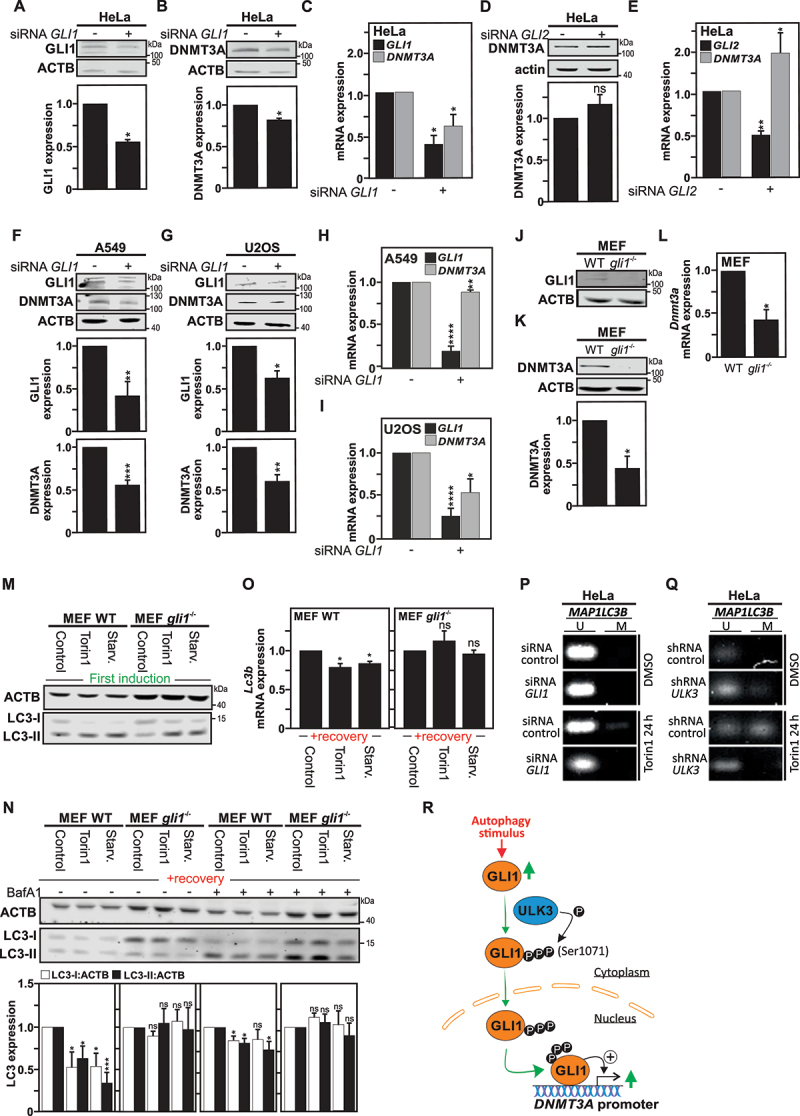
Gli1 regulates DNMT3A expression. Immunoblot analysis and quantification of GLI1 (A) and DNMT3A (B), both normalized with ACTB expression, show reduced expression for both proteins in HeLa cells transfected with *GLI1* siRNAs pools. (C) Under the same conditions, RT-qPCR analysis demonstrates reduced *GLI1* mRNA and *DNMT3A* mRNA expression in HeLa cells transfected *with GLI1* siRNAs pool. (D-E) Transfection of HeLa cells with *GLI2* siRNAs pool, despite being efficient in reducing *GLI2* mRNA expression was unable to reproduce the effects observed with *GLI1* siRNAs pool on DNMT3A expression. Immunoblot analyses (F-G) and RTqPCR analysis (H-I) and their quantifications, show similar reduction in DNMT3A expression at both protein and mRNA levels in (F, H) A549 cells and (G, I) U2OS cells transfected with the *GLI1* siRNAs pool. (J-L) likewise, *gli1^−/−^* MEF cells, deficient for GLI1 expression (J) exhibit reduced DNMT3A protein (assayed by immunoblotting, **K**), as well as *Dnmt3a* mRNA (assayed by RT-qPCR, **L**) levels as compared to WT MEF cells. (M) Immunoblot analysis for LC3-I and LC3-II expression upon either Torin1 treatment or starvation (EBSS medium) for 4 h shows occurrence of autophagy in *gli1^−/−^* and WT MEF cells as illustrated by an increase of LC3-II levels. (N) Immunoblot analysis of LC3-I and LC3-II expression in both WT and *gli1^−/−^* MEF cells either starved, treated with Torin1, or DMSO (used as control) for 4 h, thereafter, left to recover for 1 week and treated 4 h with BafA1 before harvest, showing decreased LC3 expression in WT MEF cells previously exposed to autophagy stimuli, but not in the *gli1^−/−^* MEF cells. The graph shows the quantification of LC3-I and LC3-II *versus* actin expression. (O) Reduced *MAP1LC3B* mRNA expression measured by RT-qPCR in previously autophagy-exposed WT as compared to *gli1^−/−^* MEF cells after 1 week recovery period. Methylation-specific (MS)-PCR analysis of DNA methylation level at *MAP1LC3B* locus in HeLa cells transfected either with siRNA control or siRNA *GLI1* (P) or either with shRNA control or shRNA *ULK3* (Q) treated with Torin1 for 24 h, compared with cells treated with DMSO (used as control). (R) Schematic illustration showing that autophagy induction leads to ULK3/GLI1 interaction, which promotes GLI1 activation and translocation into the nucleus. In the nucleus, GLI1 recruitment to *DNMT3A* promoter regions, promotes DNMT3A upregulation. All values are means of at least 3 independent experiments ± SEM and considered significant for *p < 0,05, **p < 0,01, ***p < 0,001 and ****p < 0,0001. n.s, not significant for the indicated comparison. (**A-L**, n = 3; **M-O**, n = 4; **P-Q**, n = 3).

Hence, we report that upon autophagy induction, GLI1 is upregulated, activated by phosphorylation by the ULK3 kinase, translocated to the nucleus and thereafter recruited to the *DNMT3A* promoter. Therefore, we wanted to explore whether targeting GLI1 expression could be enough to have an impact on *DNMT3A* gene expression. In fact, the targeting of *GLI1* gene expression in HeLa cells using a pool of small interfering RNAs (siRNAs) lead to significant downregulation of both GLI1 and DNMT3A at protein (Figure 4A-B) and mRNA levels (Figure 4C). Similar results were obtained in A549 and U2OS cells (Figure 4F-I). These results are in agreement with a previous report that the repression of *GLI1* gene expression in pancreatic cancer cells using siRNA was found to decrease *DNMT3A* gene expression [20]. Worth a notice, whereas repression of *GLI1* gene expression appeared to negatively affect *DNMT3A* gene expression, targeting the expression of *GLI2* in HeLa cells, another member of the GLI-family of transcription factor, using a pool of siRNAs was not able to recapitulate this effect (Figure 4D and E), suggesting a preferential regulation of *DNMT3A* gene expression by GLI1.

Since we previously uncovered that the induction of autophagy in cells is associated with the DNMT3A-dependent downregulation of *MAP1LC3/LC3* gene expression [18], we decided to investigate whether GLI1-deficiency can affect this process. For this purpose, we took advantage of *gli1*-deficient mouse embryonic fibroblasts (*gli1*^−/−^ MEFs), and first confirmed the absence of GLI1 expression in these cells, as well as reduced DNMT3A expression at both protein and mRNA level, as compared to wild-type MEF cells (Figure 4J-L). Furthermore, we confirmed that *gli1*^−/−^ MEFs, as wild-type MEF, respond to autophagy stimuli, such as Torin1 treatment or starvation for 4 h, as illustrated by an increased LC3-I to LC3-II conversion in these cells upon stimulation (Figure 4M). Thereafter, these cells were left to recover under normal culture conditions for 1 week and then MAP1LC3 expression was monitored at protein and mRNA levels. Whereas, *WT* MEF previously exposed to an autophagy stimulus, exhibit reduced MAP1LC3 protein expression (Figure 4N) as well as *MAP1LC3* messenger expression after a recovery period (Figure 4O), which suggest that *gli1*^−/−^ MEFs failed to acquire this cellular phenotype.

In addition, in HeLa cells, transfected with expression vector encoding wild-type ULK3, but not of the catalytically inactive ULK3^K139R^ exhibits significantly higher DNMT3A expression (**Fig. S3F-G**). In addition, whereas Torin1-treatment of mock-transfected HeLa cells lead to a significant upregulation of DNMT3A expression at both mRNA and protein levels, this failed to be observed in the catalytically inactive ULK3^K139R^-expressing cells, as well as in the HeLa transfected with the wild-type ULK3 expression vector (likely due to saturation of the system (**Fig. S3F-G**).

Finally, to further confirm a sustainable impact of GLI1 and ULK3 on the methylation of the *MAP1LC3* promoter upon autophagy induction, we made use of methylation-specific (MS)-PCR analysis, which allows the identification of DNA methylation patterns in CpG Islands. Previously, we showed that Torin1 treatment induced DNA methylation at *MAP1LC3* isoforms promoter CpG sites, which was found to be DNMT3A dependent [18]. Here, we report that the *MAP1LC3B* methylation level is found to be reduced in HeLa cells transfected with pools of siRNAs targeting *GLI1* or *ULK3* expression at 24 h post-treatment with Torin1, due to decrease of DNMT3A expression in both conditions, as compared to HeLa cells transfected with control siRNAs (Figure 4P-Q).

## Discussion

Autophagy is constitutively executed at basal level in all cells, promoting thereby cellular homeostasis. However, a growing body of evidence indicates that the autophagic activity markedly decreases with age, likely contributing to the accumulation of damaged macromolecules and organelles, as well as their associated cellular stresses, during aging. Interestingly, this decline in the efficiency of the autophagic process is also thought to contribute to age-related diseases, such as metabolic conditions, neurodegenerative diseases, cardiovascular diseases, infectious diseases, and cancer [^44–48^]. In more details, deficient autophagy has been implicated in multiple late-onset neurodegenerative diseases including amyotrophic lateral sclerosis, Alzheimer, Huntington, and Parkinson diseases, suggesting that the reported age-related impairment in neuronal autophagy leading to the accumulation of toxic protein aggregates in these cells, contributes to the onset of neurodegeneration [44,49]. Reduced autophagic activity additionally causes oxidative stress, activation of the DNA damage response, and genome instability, known causes of tumor initiation [48,50]. Furthermore, the basal housekeeping level of autophagy is considered to act as a factor of cancer suppression [23,51]. Actually, the increased incidence of cancer as people age could be linked to a decline in their underlying basal autophagy levels. Whereas the physiological decline in the autophagy efficiency in various organisms is rather well established, the mechanisms behind this biological process are yet to be fully uncovered. We recently uncovered a novel role for *de novo* DNMT3A-mediated DNA methylation in the long-term transcriptional control of *MAP1LC3* gene expression in the context of autophagy induction. Furthermore, we found that the downregulation of *MAP1LC3* gene expression, functioned as a heritable epigenetic mechanism which was associated with reduced baseline autophagy [18]. In fact, association between DNA methylation and aging is established [52]. An increase in methylation of the *Map1lc3b* promoter region as well as a decrease in its expression is reported in aged macrophages derived from 62–64 weeks mice, as compared to their young 8-week-old counterparts [53]. Downregulation of *MAP1LC3B* expression has also been reported in human rhabdomyosarcoma and medulloblastoma cancer cells transfected with a siRNA targeting GLI1 expression [54].In this present report, we bring additional understanding of the upstream molecular events that link autophagy induction to the persistent downregulation of *MAP1LC3* gene expression, with the discovery that ULK3-dependent activation of GLI1 contributes to the transcriptional upregulation of *DNMT3A* gene expression upon autophagy induction. In addition, our study uncovers a novel role of ULK3 upon autophagy induction and contribution to GLI1-mediated DNMT3A DNA methylation (Figure 4R).

However, further investigations will be required to establish whether the ULK3-GLI1-DNMT3A-MAP1LC3 axis we uncovered is involved in the decline of the housekeeping function of autophagy observed during aging and a predisposition to age-related diseases.

## Material and methods

### Reagents and antibodies

Reagents used in this study include bafilomycin A_1_ (BafA1) (Santa Cruz Biotechnology, sc-201,550), carbamazepine (CBZ) (Sigma-Aldrich, C4024), clonidine (Sigma-Aldrich, C7898), Hoechst 33,342 (Molecular probes/Invitrogen, H3570), torin1 (Tocris, 4247), and trehalose (Sigma-Aldrich, T9531).

Primary antibodies used in this study include ACTB (mouse monoclonal antibody [mAb]; Sigma Aldrich, A-3853), ATG5 (mouse mAb; Bio-techne, 603,813), ATG7 (rabbit polyclonal antibody [pAb]; Genetex, GTX61647), DNMT3A (rabbit pAb; Abcam, ab2850 and Santa Cruz Biotechnology, sc20703), FLAG (rabbit pAb; Abcam, ab1162), GLI1 (rabbit pAb; Novus Biologicals, N-600-600), LMNB1 (rabbit pAb; Abcam, ab16048), LC3B (rabbit pAb; Sigma Life Science, L7543), phosphoserine, clone 4A4 (mouse mAb; Milipore (05–1000), phospho-GLI1(Ser1071) (rabbit pAb; Fisher Scientific, PA5105346), TUBA1A (mouse mAb; Cell signaling, 3873S), ULK3 (mouse mAb; Biocompare, MBS9200567).

### Cell culture, treatments, and transfections

Human cervical carcinoma HeLa cells (ATCC®, CCL-2™) were freshly obtained from Sigma and were grown with Minimum Essential Medium (MEM; Gibco®, 21,090,022), 10% fetal bovine serum (FBS; Gibco®, 10,270,106), 1% L-glutamine (Gibco®, 25,030,024) and 1% penicillin-streptomycin (Gibco®, 10,478,016) and supplemented with 1% sodium pyruvate (Gibco®, 11,360,070) and 1% MEM non-essential amino acids (Gibco®, 11,140,050). A549 (ATCC®, CCL-185™) and U2OS (ATCC®, HTB-96™) were grown with RPMI 1640 medium (Gibco®, 11,875,093), 10% FBS, 1% L-glutamine and 1% penicillin-streptomycin. Mouse embryonic fibroblasts (*WT* MEF from ATCC®, CRL-2214™; *gli1^−/−^* MEFs were kind gift from Dr Rune Toftgård, Karolinska Institutet) cells were grown with Dulbecco’s Modified Eagle’s Medium (DMEM; Gibco 61,965,026), 10% FBS, 1% L-glutamine and 1% penicillin-streptomycin. Cells were maintained under normal cell culture conditions in a humidified atmosphere at 37°C degrees and 5% CO_2_. HeLa, A549 and U2OS cell lines were seed in 6 well-plates up to 50% confluence. Cells were transfected with Lipofectamine 3000 (Invitrogen, L3000015) and either non-targeting control siRNA or targeted siRNA as *GLI1, GLI2, ULK1, ULK2, ATG5* or *ATG7* ON-TARGET plus SMARTpools siRNA (All from Dharmacon™), whose siRNA sequences can be found in **Table S1**. Analysis was performed 48 h after transfection. siRNA-mediated knockdown was either confirmed by immunoblotting or RT-qPCR. HeLa cells were transfected with hGli1 FLAG 3x, which was a gift from Dr Martin Fernandez-Zapico (Addgene, 84,922; http://n2t.net/addgene:84922; RRID:Addgene_84,922) and/or with with FLAG-tagged constructs expressing ULK3 WT or ULK3^K139R^ kinase mutant were kindly provided by Dr. Alla Piirsoo (University of Tartu, Estonia).

### RNA extraction, cDNA synthesis and real-time quantitative PCR

HeLa cells were seeded in 6-well plates and subjected to the corresponding treatment or transfection. After the indicated timepoint, RNA was extracted following the RNeasy Mini Kit (Qiagen, 74,104) manufacturer’s instructions. RNA concentrations were determined using the NanoDrop® spectrophotometer (Thermo Fisher Scientific). One µg RNA using oligo dT, dNTPs, and Superscript IV Reverse Transcriptase (Invitrogen, 18,090,200) was used for cDNA synthesis. qPCR was run on a Step One (Applied Biosystems) using SYBR™ Green master mix (Qiagen, 4,385,610). For normalization and analysis, *GAPDH* and *ACTB* were used as housekeeping genes. The specific primers sequence used in the study can be found in the **Table S2**. Results were calculated using the delta Ct method and represented as a fold change over untreated cells or transfected with Control siRNA with the treated ones. Further analysis and statistical evaluation were performed using the R data analysis software (Bioconductor package).

### Immunoblotting

Total protein extracts were obtained using 5x Laemmli buffer (62.5 mM Tris-HCl, pH 6.8, 2% SDS, 10% glycerol, 5% β-mercaptoethanol, 0.02% bromophenol blue). For immunoblot analysis, protein extracts were sonicated, boiled at 96°C for 8 min. Proteins were resolved in 8% or 15% SDS–polyacrylamide gel electrophoresis and blotted onto 0.2-µm or 0.45-µm pore-size nitrocellulose membranes in a wet transfer system. Membranes were blocked for 1 h either with 5% bovine serum albumin (BSA; Sigma-Aldrich, A9418) in Tris buffered saline (20 mM Tris-base, pH 7.6, 150 mM NaCl) with 1% Tween 20 (Sigma-Aldrich, P1379; TBS-T) or 5% milk in phosphate-buffered saline (PBS; Gibco®, SH300028.02) with 1% Tween 20 (PBS-T) buffer. For protein detection, membranes were incubated overnight at 4°C with the corresponding primary antibody (listed above). The day after, membranes were washed 3 times with PBS-T and incubated with the appropriate secondary antibody, RDye® 680RD Goat anti-Rabbit or RDye® 800CW Goat anti-Mouse IgG (1:5000; LI-COR Bioscience, 926–68,071 and 926–32,210 respectively) for 1 h at room temperature. Visualization was obtained using an Odyssey CLx infrared imaging system (LI-COR Bioscience). Analysis was made using anti-*ACTB/β-actin* as housekeeping gene for standardization of protein loading, and densitometry was done using the ImageJ software normalizing the protein of interest to loading control. Both, in the same linear range of acquisition.

### Subcellular fractionation

Cytoplasmic and nuclear protein fractions were collected using the NE-PER^TM^ Nuclear and Cytoplasmic Extraction Reagents (ThermoFisher Scientific, 78,835) following the manufacturer’s instructions. After subcellular fraction purification, the samples were resolved by SDS-PAGE and analyzed by immunobloting as previously described.

### *ULK3* gene silencing by shRNA lentiviral infection in Hela cells

ShRNA for *ULK3* in pLKO-PURO lentiviral particles were purchased from Sigma Aldrich. Two clones were tested by lentiviral infection (MOI 5) of the HeLa cells overnight in the presence of polybrene (Sigma-Aldrich, TR-1003). After 2 days, cells were selected using fresh medium containing 1 µg/ml of puromycin (Sigma-Aldrich, P9620) and shRNA efficiency was monitored by Western blotting analysis (as previously described) over the time following the infection and after few cell passages.

### Immunofluorescence

For confocal microscopy analysis, the adherent mammalian cells were grown on coverslips for 24 h and treated with the corresponding reagent. Cells were fixed with 4% paraformaldehyde for 15 min at room temperature, and blocked in HEPES buffer (10 mM HEPES, pH 7.2 [Sigma-Aldrich, H3375], 3% bovine serum albumin, 0.3% Triton X-100 [Sigma-Aldrich, T8787] diluted in PBS) at room temperature during 1 h. Later, the slides were left overnight in a humidified chamber at 4°C with the indicated primary antibody. The day after, the samples were washed 3 times with 0.1 Tween-PBS for 5 min and incubated with the corresponding secondary antibody Alexa Fluor® 488 goat anti-rabbit IgGs or Alexa Fluor® 488 goat anti-mouse (1:500; Molecular Probes/Invitrogen, A11008 and A11029, respectively) at room temperature for 1 h. Antibodies are listed in section above. Slides were mounted using ProLong^TM^ Gold antifade Mounting medium with DAPI (Molecular probes/Invitrogen, P36931) used to stain the nuclei. Samples were analyzed under Zeiss LSM700 confocal laser scanning microscopy equipped with ZEN Zeiss software.

### In-situ proximity ligation assay (PLA)

HeLa cells were grown on coverslips and fixed with 3.7% paraformaldehyde in PBS for 15 min. The *In-situ* PLA (Duolink II Detection Reagents Red Kit; Olink Bioscience, DUO92101) was performed according to manufacturer’s instruction. Before the last step, the samples were dried for approximately 10 min under the hood at room temperature protected from light and mounted using ProLong Gold antifade mounting medium containing DAPI. Fluorescence signal amplification was observed using the Zeiss LSM700 confocal laser scanning microscopy. Antibodies used are listed above.

## Chromatin immunoprecipitation (ChIP)

ChIP experiments were done using the HighCell# ChIP kit from Diagenode (kch-mahigh-G48) following the manufacturer´s instructions. Briefly, cells were cross-linked in 1% formaldehyde following cell lysis. Next, chromatin shearing was done using a Bioruptor® Pico sonicator (Diagenode). Magnetic beads and 6.3 μg of antibody were incubated for 4 h at 4°C to promote successful binding following an overnight incubation of beads-antibody-sheared chromatin at 4°C. Normal rabbit IgGs (R&D systems, AB-105-C) were used as negative controls. 1% input of purified DNA were then analyzed by qPCR (The sequences of the primers used for ChIP are listed in **Table S2**). Data interpretation from qPCR was done by calculation of the percentage to input and then normalized to control conditions.

### Analysis of methylated DNA by MS-PCR

HeLa cells were seed in 6 well-plates and transfected either with siRNA Control or siRNA against *GLI1*. The following day, cells were treated with DMSO used as control or Torin1 for 24 h in HeLa cells. Cells were harvested after 48 h of transfection. Similarly, shControl and sh*ULK3* HeLa cells were seed in 6-well plates and treated either with DMSO or Torin1 for 24 h. Thereafter, cells were harvested and subjected to further analysis. Lysis and bisulfite conversion were carried out using the EpiTect Fast lyseAll Bisulfite kit (Qiagen, 59,864) and following the manufacturer´s instructions. Converted DNA (1 µL) was used for PCR using the HotStarTaq Master Mix (Qiagen, 203,443). PCR was run in a T100™ Thermal Cycler (Bio-Rad, Hercules, CA, USA) at 56°C for 34 cycles using the corresponding MS-PCR primers previously used [18]. The PCR products from the Bisulfite converted samples were run on a 2% agarose gel containing Sybr safe (Invitrogen, S33102) and revealed using a Gel Doc™ EZ Imager (Bio-Rad).

### Statistical analyses

Statistical differences were perform using Student’s t-test; *p < 0.05, ** p < 0.01, *** p < 0.001, **** p < 0.0001. n.s., not significant for indicated comparison. Error bars indicate standard error mean (SEM) of at least three independent experiments.

## Supplementary Material

Supplemental Material
