# Supplementary material for: ULK3-dependent activation of GLI1 promotes DNMT3A expression upon autophagy induction
Source: Autophagy. 2022 Feb 28;18(12):2769–80. doi: 10.1080/15548627.2022.2039993 (PMC9673947; doi:10.1080/15548627.2022.2039993)
Supplement: Supplemental Material [file KAUP_A_2039993_SM6217.docx]

**Supplementary Material file**

**ULK3-dependent activation of GLI1 promotes DNMT3A expression upon autophagy induction**

Supplementary Figure S1 to S3

Supplementary Table S1 to S2

**
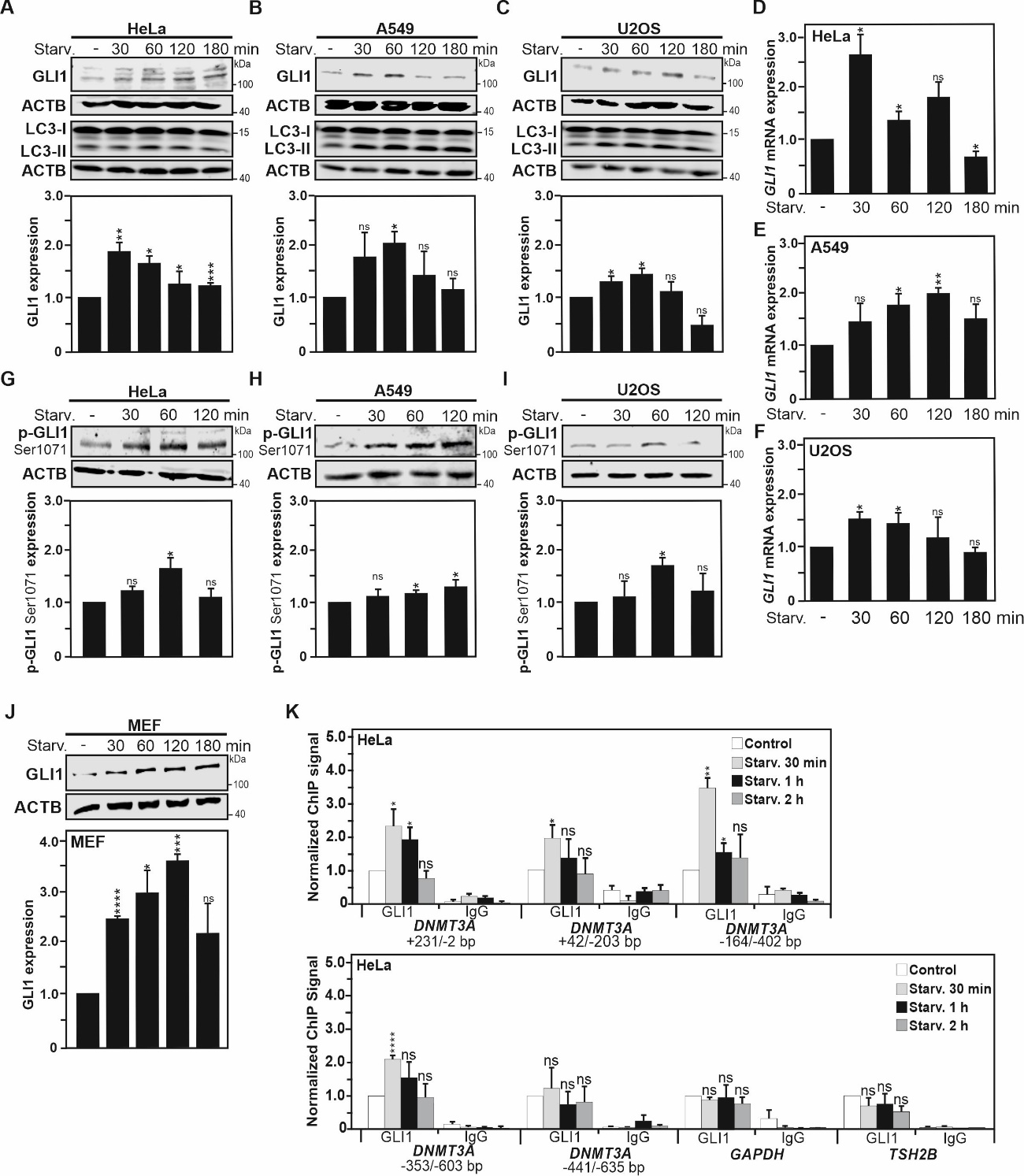
**

**Figure S1.** Starvation led to GLI1 upregulation, phosphorylation and recruitment to *DNMT3A* promotor. Immunoblot analysis of GLI1, LC3-I and LC3-II expression in (**A**) HeLa cells, (**B**) A549 cells and (**C**) U2OS cells upon nutrient starvation at 30-, 60-, 120- and 180-min timepoints reveal an increase in GLI1 protein expression upon autophagy induction. (A-C) The graphs show quantification of GLI1 versus ACTB expression in respective cell lines. RT-qPCR analysis of *Gli1* mRNA expression in (**D**) HeLa cells, (**E**) A549 cells and (**F**) U2OS cells upon same treatment as above. Analysis of GLI1 phosphorylation at serine 1071 residue (p-GLI1 Ser1071) by immunoblotting after 30-, 60-, and 120-min starvation indicates increased GLI1 phosphorylation upon autophagy induction in (**G**) HeLa cells, (**H**) A549 cells and (**I**) U2OS cells. (G-H) The graphs display the quantification of p-GLI1 Ser1071 versus ACTB expression. (**J**) Immunoblot analysis and quantification of GLI1 expression in MEF cells upon starvation at 30, 60, 120 and 180 min reveal an increase in GLI1 protein expression upon autophagy induction. (**K**) ChIP analysis of GLI1 recruitment on *DNMT3A* promoter and exon 1 regions (depicted in panel E of figure 1) upon induction of autophagy with starvation in HeLa cells show transient GLI1 enrichment at 30 min and/or 1 h time point at +231/-2 bp, +42/-203 bp, -164/-402 bp and -353/-603 bp. No significant GLI1 enrichments were observed at *GAPDH* and *TSH2B* loci used as negative controls. All values are means of at least 3 independent experiments ± SEM and considered significant for *p<0,05, **p<0,01, ***p<0,001 and ****p<0,0001. n.s, not significant for the indicated comparison. (**A,** n=3; **B**, n=3; **C**, n=4; **D-F**, n=3; **G-I**, n=4; **J**, n=3; **K**, n=3).

**
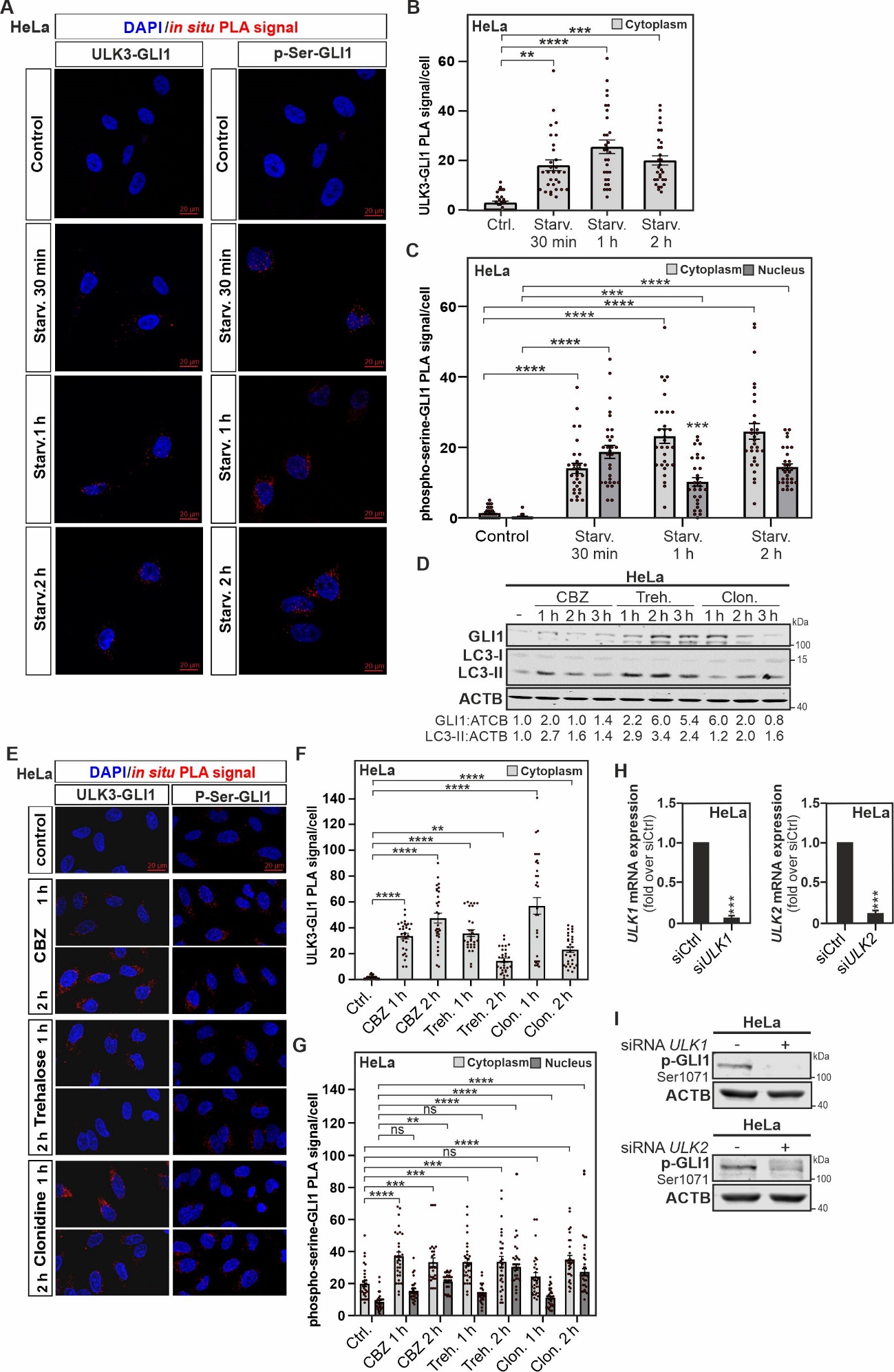
**

**Figure S2.** GLI1 is upregulated and phosphorylated by ULK3 upon autophagy induction with starvation or MTOR-independent inducers. (**A**) *in situ* PLA assays were performed to assess ULK3-GLI1 interaction and phosphorylation of GLI1 at serine residues upon starvation at 30 min, 1 or 2 h showing that GLI1 interacts with ULK3, is phosphorylated at serine residues and shuttled to the nucleus in HeLa cells upon starvation. Scale: 20 µm. Statistical analysis of ULK3-GLI1 interactions per cell shows significant increase already at 30 min, (**B**) and phospho-serine-GLI1 interaction at the cytoplasm and the nucleus as early as 30 min and sustained for up to 2 h upon starvation (**C**). Statistics were performed with a one-way ANOVA, n=30 and 95% confidence intervals are shown. (**D**) Treatment with carbamazepine (CBZ), trehalose (Treh.) or clonidine (Clon.) in HeLa cells shows an increase of GLI1 expression that correlates with LC3-II accumulation in HeLa cells. (**E**) *in situ* PLA assays as described in panel A were performed on HeLa cells treated with carbamazepine, trehalose or clonidine for 1 or 2 h. Scale: 20 µm. Quantification of the *in situ* PLA displayed on panel E shows an increase in the number of (**F**) ULK3-GLI1 interactions as well as (**G**) an increases of phospho-serine-GLI1 interactions per cell at 1 or 2 h post treatment with different MTOR-independent inducers of autophagy. (**H**) RT-qPCR analysis of *Ulk1 and Ulk2* mRNA expression in HeLa cells transfected with siRNA pools designed to target *Ulk1 and Ulk2.* Scramble control siRNA was used as control. (**I**) Analysis of GLI1 phosphorylation at serine 1071 residue (p-GLI1 Ser1071) by immunoblotting indicates decreased GLI1 phosphorylation in *Ulk1-*siRNA and *Ulk2*-siRNA transfected HeLa cells. Statistics were performed with a one-way or two-way ANOVA respectively (Pairwise Multiple Comparison by the Holm-Sidak method), n=30 and 95% confidence intervals are shown. All values are means of at least 3 independent experiments ± SEM and considered significant for *p<0,05, **p<0,01, ***p<0,001 and ****p<0,0001. n.s, not significant for the indicated comparison. (**A,** n=3; **B**, n=3; **C**, n=4; **D-F**, n=3; **G-I**, n=4; **J**, n=3; **K**, n=3; **H**=3; **I**=3).

**
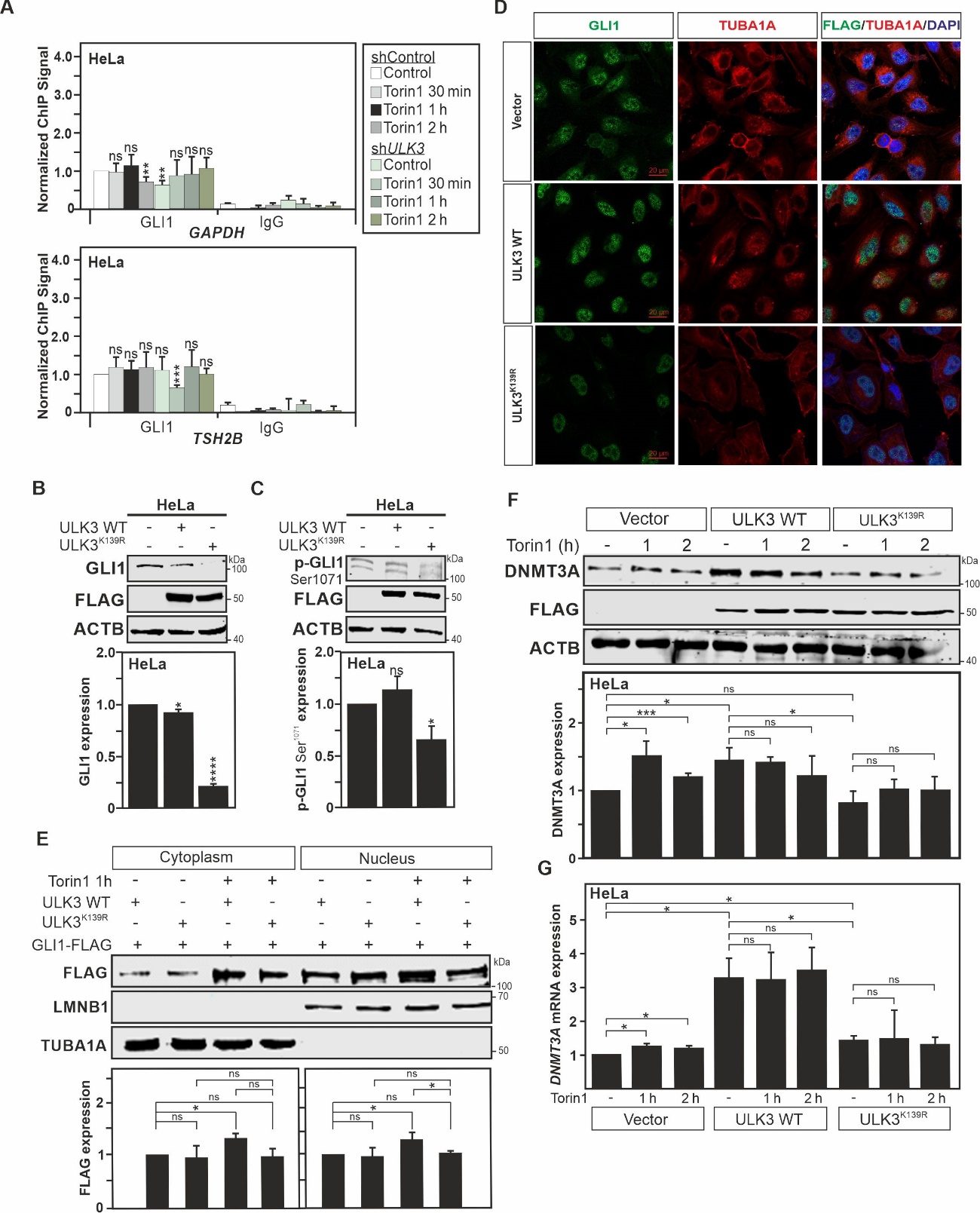
**

**Figure S3.** ULK3 kinase activity mediates GLI1 activation, translocation into the nucleus and enhances DNMT3A expression. (**A**) ChIP analysis of GLI1 occupancy at *GAPDH* and *TSH2* locus in shRNA *ULK3* and shRNA control Hela cells, treated with Torin1, for 30 min, 1 and 2 h showing no GLI1 enrichment (supplementary data to panels I and J of figure 3). Immunoblot analysis of GLI1 (**B**) and p-GLI1 Ser1071 (**C**) expression levels in HeLa cells transfected either with an empty plasmid, a Flag-tagged versions of WT ULK3, or for a point mutated ULK3^K139R^, catalytically inactive version of ULK3 revealed the importance of ULK3 kinase activity in GLI1 regulation. (**D**) Immunofluorescence analysis of GLI1 and TUBA1 expression in HeLa cells transfected as described in panel B. DAPI was used for nuclear counterstaining. Scale: 20 µm. (**E**) Immunoblot analysis of cytoplasmic and nuclear fractions collected from HeLa cells co-transfected with FLAG-tagged GLI1 and ULK3-WT or ULK3^K139R^ expression vectors for 24 h and thereafter treated with Torin1 for 1 h demonstrating an increase in nuclear localization of GLI1 in ULK3-WT but not ULK3^K139R^-expressing HeLa cells. The graph shows quantification of expression for FLAG *versus* TUBA1A (used as cytosolic marker) and FLAG *versus* LMNB1 (used as nuclear marker). (**F-G**) Analysis of DNMT3A protein (by immunoblot) and mRNA (by RT-qPCR) expression levels upon transfection of HeLa cells as described in panel E and thereafter treatment with Torin1 for 1 and 2 h. All values are means of at least 3 independent experiments ± SEM and considered significant for *p<0,05, **p<0,01, ***p<0,001 and ****p<0,0001. n.s, not significant for the indicated comparison. (**A,** n=3; **B-C**, n=4; **D**, n=3; **E**, n=3; **F-G**, n=4).

**Table S1**. List of the sequences of the small interfering RNAs used in this study.

| **ON-TARGET plus SMARTpools siRNAs** | **Companies** | |
| --- | --- | --- |
|  |  | |
| ***ATG5*** (human, ATG5 NM_004849) |  | |
| GGCAUUAUCCAAUUGGUUU | Dharmacon (L-020112) | |
| GCAGAACCAUACUAUUUGC |  | |
| UGACAGAUUUGACCAGUUU |  | |
| ACAAAGAUGUGCUUCGAGA |  | |
|  |  | |
| ***ATG7*** (human, *ATG7* NM_006395) |  | |
| CCAACACACUCGAGUCUUU | Dharmacon (L-020112) | |
| GAUCUAAAUCUCAAACUGA |  | |
| GCCCACAGAUGGAGUAGCA |  | |
| GCCAGAGGAUUCAACAUGA |  | |
|  |  | |
| ***GLI1*** (human, GLI1 NM_001167609) |  | |
| GCAAAUAGGGCUUCACAUA | Dharmacon (L-003896) | |
| AGGCUCAGCUUGUGUGUAA |  | |
| GGACGAGGGACCUUGCAUU |  | |
| CAGCUAGAGUCCAGAGGUU |  | |
| ***GLI2*** (human, GLI2 NM_001371271)  CGUCAACCCUGUCGCCAUU  AGGCUGAGGUGGUCAUCUA  GCACACCGCUGCUCAAAGA  UCAAGUCACUCAAGGAUUC | Dharmacon (L-006468) | |
| ***ULK1*** (human, ULK1 NM_003565)  CAGCAUCACUGCCGAGAGG  CCACGCAGGUGCAGAACUA  GCACAGAGACCGUGGGCAA | Dharmacon (L-005049) | |
| UCACUGACCUGCUCCUUAA  ***ULK2*** (human, ULK2 NM_001142610)  GUGGAGACCUCGCAGAUUA  UCAGACCACUCAUGUGAUA  CGAUAUAAAUUCUGCAUCA  UCCAAGAUCUGCAGUGGUA | | Dharmacon (L-005396) |
| **Non-targeting siRNA pool** |  | |
| UGGUUUACAUGUCGACUAA | Dharmacon (D-001810) | |
| UGGUUUACAUGUUGUGUGA |  | |
| UGGUUUACAUGUUUUCUGA |  | |
| UGGUUUACAUGUUUUCCUA |  | |
|  |  | |

**Table S2**. List of the primer sequences used. PCR primers for quantitation of mRNA levels, ChIP or MS-PCR. All the sequences are given 5’ to 3’.

| **cDNA** (organism) | **Forward Primer** | **Reverse Primer** |
| --- | --- | --- |
| **Primers used for qPCR** | | |
| ***ACTB***  (human) | gatcaagatcattgctcctc | ttgtcaagaaagggtgtaac |
| ***DNMT3A***  (human) | gaagagaagaatccctacaaag | caataatctccttgaccttgg |
| ***Dnmt3a***  (mouse) | ggcatggactgtggtcatgag | caatgatctccttgacctag |
| ***GAPDH***  (human) | acagttgccatgtagacc | tttttggttgagcacagg |
| ***Gapdh***  (mouse) | tgcaccaccaattgcttagc | ggcatggactgtggtcatgag |
| ***GLI1***  (human) | agatgaatcaccaaaaaggg | atatcaccttccaagggttc |
| ***GLI2***  (human) | taccagcagattctgagc | ctctgcttgttctggttg |
| ***Map1lc3b***  (mouse) | gctcatcaagataatcagacg | gcataaaccatgtacaggaag |
| ***ULK1***  (human) | tcaaaatcctgaaggaactg | accaggtagacagaattagc |
| ***ULK2***  (human) | atggctcctgaggttattatg | gcattggtatatcactgttcc |
| **Primers used for ChIP** | | |
| ChIP ***DNMT3A* -635/-441**  (human) | gccatgtcctgtgccagtca | ctcactatgtgctcatctcactcct |
| ChIP ***DNMT3A* -603/-353**  (human) | cactgtgatatagctgaagtgctg | gtgggggctgttctcctt |
| ChIP ***DNMT3A* -402/-164**  (human) | aggaacctagagccctgagc | caggctccaaagcctctct |
| ChIP ***DNMT3A* -203/+42**  (human) | aggtacggggaactcactcc | cttcgccctgcagttctc |
| ChIP ***DNMT3A* -203/+42**  (human) | gtgctgaggcaggcagag | cagagcccctcgagtcgt |
| ChIP ***GAPDH*** | Human ChIP-seq grade GAPDH TSS primer pair | |
| (human) | (Diagenode; C17011047); Sequence under disclosure | |
| ChIP ***TSH2B*** | Human ChIP-seq grade TSH2B promoter primer pair | |
| (human) | (Diagenode; C17011041); Sequence under disclosure | |
| **Primers used for MS-PCR** |  |  |
| **Methylated *MAP1LC3B*** | gtttagaatgaaggttcggga | ataaccgctaataacctccgc |
| (human) |  |  |
| **Unmethylated** ***MAP1LC3B*** | gtttagaatgaaggtttggga | aaataaccactaataacctccaccc |
| (human) |  |  |
|  |  |  |
|  |  |  |
